# Direct observation of melting in a two-dimensional driven granular system

**DOI:** 10.1038/srep24056

**Published:** 2016-04-07

**Authors:** Xiaoyan Sun, Yang Li, Yuqiang Ma, Zexin Zhang

**Affiliations:** 1 Centre for Soft Condensed Matter Physics and Interdisciplinary Research, Soochow University, Suzhou 215006, China; 2 National Laboratory of Solid State Microstructures and Department of Physics, Nanjing University, Nanjing 210093, China; 3 Kavli Institute for Theoretical Physics China, CAS, Beijing 100190, China

## Abstract

Melting is considered to be one of the most fundamental problems in physical science. Generally, dimensionality plays an important role in melting. In three-dimension, it’s well known that a crystal melts directly into a liquid via a first-order transition. In two-dimension (2D), however, the melting process has been widely debated whether it is a first-order transition or a two-step transition with an intermediate hexatic phase. Experimentally 2D melting has been intensively studied in equilibrium systems such as molecular and colloidal crystals, but rarely been explored in non-equilibrium system such as granular materials. In this paper, we experimentally studied the 2D melting in a driven granular model system at single particle level using video recording and particle tracking techniques. Measurements of orientational/translational correlation functions show evidences that the melting is a two-step transition. A novel concept of orientational/translational susceptibilities enable us to clearly resolve the intermediate hexatic phase. Our results are in excellent agreement with the two-step melting scenario predicted by KTHNY theory, and demonstrate that the KTHNY melting scenario can be extended to non-equilibrium systems.

Over the decades, two-dimensional melting has been widely debated whether it undergoes a first-order transition^1^,^2^,^3^,^4^,^5^ (directly from crystal to a liquid phase), or a two-step transition^6^,^7^,^8^,^9^,^10^,^11^ (first from crystal to a hexatic and then from the hexatic to liquid), as predicted by Kosterlitz-Thouless-Halperin-Nelson-Young (KTHNY theory). The intermediate hexatic phase has kept the quasi-long-range orientational order found in crystal, but changes the translational order from long-range to short-range. The two transitions are also characterized by the binding and unbinding of topological defects, i.e., dislocations and disclinations, respectively. These features exist in the experiments across a wide range of systems, including molecules^12^ and electrons^9^, colloidal suspensions^13^,^14^,^15^, and superconductors^16^. Although the results of many colloidal experiments and computer simulations are consistent with KTHNY theory^17^,^18^,^19^,^20^,^21^, others provide strong evidences to support a first-order transition^5^. So it is highly desirable to explore 2D melting in other model systems.

In this paper, we experimentally studied the nature of 2D melting using an air-fluidized model granular system^22^,^23^. This driven granular system, though it is far from equilibrium, has shown strong similarities to equilibrium systems, and has successfully modelled jamming and glass transitions^24^,^25^,^26^. In a related vein, a vibrated granular system has been used to study 2D melting by varying the intensity of the vibration. However, it has a limited parameter space of three vibration amplitudes^27^. Herein, we systemically varied the packing fraction of an air-fluidized granular system to investigate the nature of the 2D melting. We measured a variety of sample properties during the melting process, including pair correlation function, static structure factor, orientational/translational order correlation functions, orientational/translational susceptibilities, dynamic Lindemann parameter and defect dynamics. By orientational/translational order correlation functions, we observed two-step melting transition. We then applied a novel concept of the orientational/translational susceptibilities, which has never been used in granular systems, to analyze the melting, and clearly resolved the intermediate hexatic phase. In addition, real-space images from the experiments revealed that the unbinding of dislocations drove the crystal to the hexatic phase and the unbinding of disclinations drove the hexatic phase to the liquid. Our results showed that the 2D melting behaviours of driven, non-equilibrium granular systems are in agreement with computer simulations of 2D melting in equilibrium system^16^,^22^, which follows KTHNY theory with a two-step phase transition and an intermediate hexatic phase.

## Results and Discussions

### Static Structure

**Figure 1:**
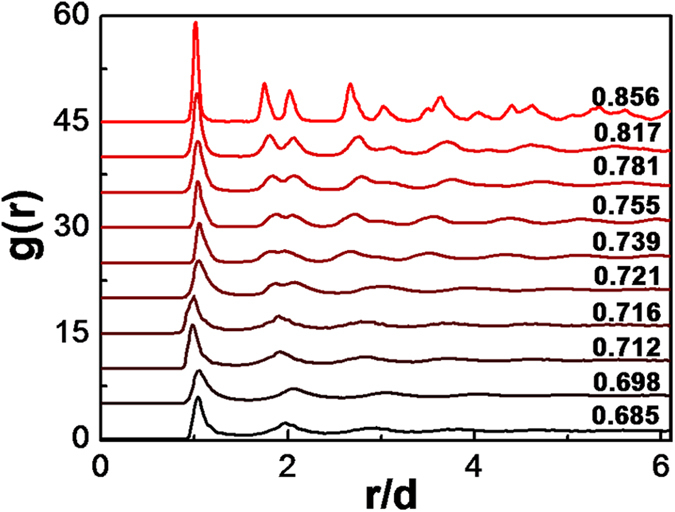
Pair correlation function g(r) at different packing fractions. As the packing fraction, ϕ decreases from 0.856 to 0.685, the height of the first peak decreased at hard core contact r/d ≈ 1, and the multi-peaks in the long-range parts gradually disappear. The curves are shifted vertically for clarity.

To characterize the static structure of the granular system during 2D melting, we calculate pair correlation function, g(r)^27^, and 2D structure factor, S(k, t)^28^. The pair correlation function is defined as


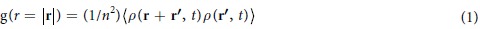


where 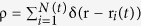 is the distribution of particles in the field of view and *n* is the number density of the particles. The angular brackets denote an average over time and space. We can see from Fig. 1 that, as the packing fraction, ϕ decreases, the pair correlation functions show less and less regularly spaced peaks, indicating the system melts from an ordered crystal to a disordered liquid.

**Figure 2:**
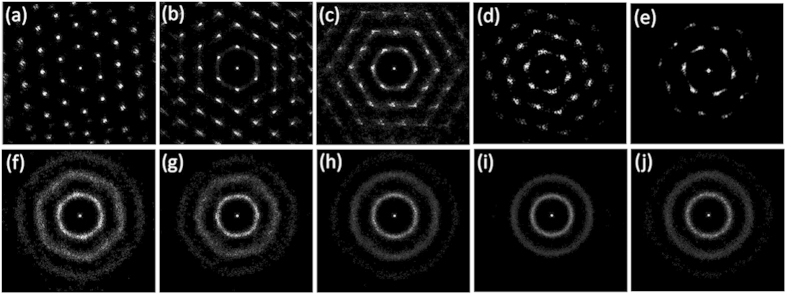
2D structure factors for different packing fractions. From (**a**–**j**), the packing fraction, ϕ is decreasing, with ϕ = 0.856, 0.817, 0.781, 0.755, 0.739, 0.721, 0.716, 0.712, 0.698, and 0.685 respectively. The discrete bright dots in a hexagonal array shows a crystal phase (panels (**a**,**b**)), and the bright rings shows a liquid phase (panels (**h**–**j**)).

The 2D structure factor, S(k, t) is obtained by Fourier transforming of pair correlation function, g(r). Snapshots of 2D structure factor, S(k, t) are presented in Fig. 2. The patterns are similar with X-ray or electron diffraction from molecular 2D systems. For the crystal phase, the patterns are discrete bright spots in a hexagonal array [Fig. 2(a,b)]. For the liquid phase, the dots vanish with diffusive rings left [Fig. 2(h–j)]. And for the hexatic phase, the 2D structure factor, S(k, t) shows a blending of bright spots and the diffusive rings [Fig. 2(c–g)].

### Order parameter correlations in space and time

To better resolve the 2D melting, we turn to analyze other correlation functions, namely orientational correlation function and translational correlation function, which are more sensitive to the structural order than pair correlation function and structure factor. According to the KTHNY theory, different phases can be directly characterized by the orientational/translational correlation functions^11^:


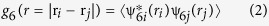



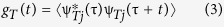


where *ψ*_6_(*r*) and *ψ*_T_(*t*) are the local orientational and translational order parameters of the particle *i* at the position *r*_*i*_ *respectively*. Here for particle *j*, the orientational order parameter, *ψ*_6_(*r*), is


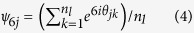


where *n*_*j*_ is the number of the nearest neighbour of particle *j* and *θ*_jk_ is the angle of the bond between particle *j* and its neighbours *k*. The translational order parameter, *ψ*_T_(*t*), is defined:


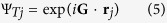


where **G** is a primary reciprocal lattice vector, and **r**_*j*_ = (*x*_*j*_*, y*_*j*_).

**Figure 3:**
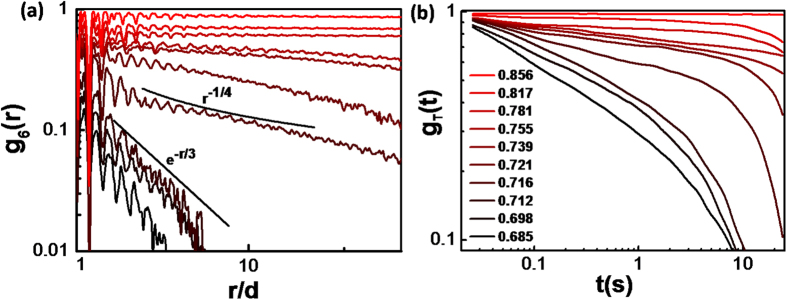
Orientational/Translational correlation functions for different packing fractions. (**a**) Orientational correlation function g_6_(r) in a semi-logarithmic plot. The solid lines are the power law decay (*r*^−1/4^) and exponential decay (e^−1/3^). The *r*^−1/4^ decaying behaviour at the hexatic-liquid transition is predicted by KTHNY theory. (**b**) Translational correlation function g_T_(t) in a log-log plot. The legends apply to both panels.

Orientational correlation function can be used to semi-quantitatively distinguish three regimes corresponding to crystal, hexatic and liquid phases as predicted by KTHNY theory [Fig. 3(a)]. For the high packing fractions (ϕ > 0.781), the orientational correlation function *g*_6_(*r*) is nearly constant, indicating the system is in a crystal phase with long-range orientational order. For the intermediate packing fractions (ϕ = 0.716 ~ 0.781), the orientational correlation function *g*_6_(*r*) shows a pow-law decaying behaviour with 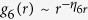, suggesting that the system is in the hexatic phase with quasi-long-range orientational order.^11^,^14^,^28^,^29^,^30^,^31^ And for the low packing fractions (ϕ < 0.716), the orientational correlation functions *g*_6_(*r*) decays exponentially, the system becomes a liquid. Note near the hexatic-liquid transition point (ϕ = 0.716), *η* is close to 1/4, which agrees with the power law decay of *g*_6_(*r*) as predicted by the KTHNY theory (note *η* = 1/4 is the hexatic-liquid transition point). The translational correlation *g*_T_(*t*) yields consistent results, with a slow decay of 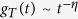 for crystal phase (ϕ > 0.781), and a fast decay of 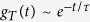 for hexatic and liquid phases (ϕ < 0.781) [Fig. 3(b)].

### Susceptibilities

Although the pair correlation function, structure factors, and correlation functions have been widely used to analyse the 2D melting, there are finite-size or finite-time ambiguities in these analyses. For example, at finite time scale, the correlation functions exhibit a power-law decay, but at longer times, it may decay exponentially^11^,^32^,^33^. So in order to ameliorate these ambiguities, we explored a new physics quantity, the order parameter susceptibility^11^, to define the transition points. The order parameter susceptibility is defined as:


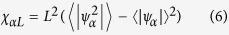


Here L is the system size, 
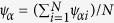
is the average value of the order parameter over all N particles in the box with the size of *L* × *L* and α = 6, T. To ameliorate finite-size effects, we calculate *χ*_*L*_ in multiple sub-boxes containing different numbers of particles, and then extrapolate it to the infinite size limit (see the Supplementary Material for details of the extrapolation method).

**Figure 4:**
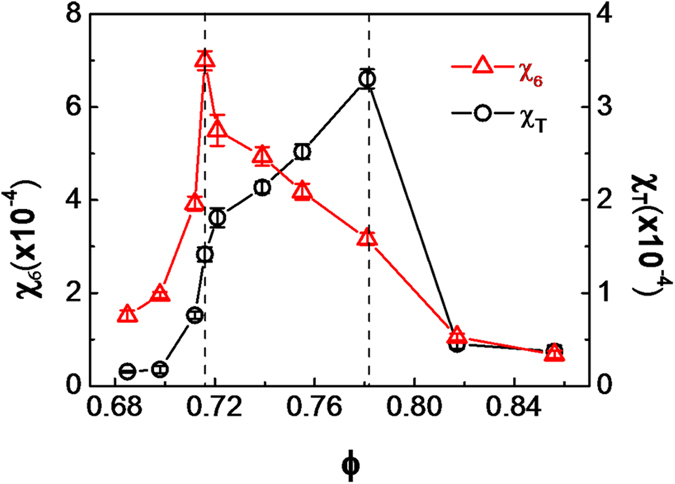
The susceptibilities, χ_6_ and χ_T_ as a function of packing fractions. The peaks of the orientational susceptibility χ_6_ and the translational susceptibility χ_T_ clearly indicate two transition points, at ϕ = 0.716 and ϕ = 0.781 respectively. The error bars are standard deviations from three independent calculations and extrapolations.

The results of the susceptibilities χ_T_ and χ_6_ are shown in Fig. 4. The two sharp peaks of the susceptibilities can unambiguously indicate two transitions in the 2D melting. The susceptibility of the translational order parameter χ_T_ diverges at ϕ = 0.781, indicating the transition from the solid to the hetaxic phase; and the orientational order parameter χ_6_ diverges at ϕ = 0.716, indicating the transition from the hetaxic to the liquid phase. So the packing fraction from 0.716 to 0.781 is the hexatic phase, which is consistent with the results obtained from the pair correlation function, structure factor and orientational/translational correlation functions.

### Dynamic Lindermann parameter

The dynamical behavior of the system can be characterized by the Lindermann parameter. The traditional Lindemann parameter diverges in 2D because of the strong long-wavelength fluctuations^34^. Hence for 2D systems, a dynamic Lindemann parameter^11^,^34^ is defined as


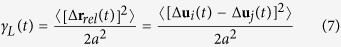


where 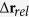 is the displacement of relative nearest neighbor-neighbor, Δ**u**_*i*_ is the displacement of particle *i*, also particles *i* and *j* are nearest neighbors, and a is the lattice constant.

**Figure 5:**
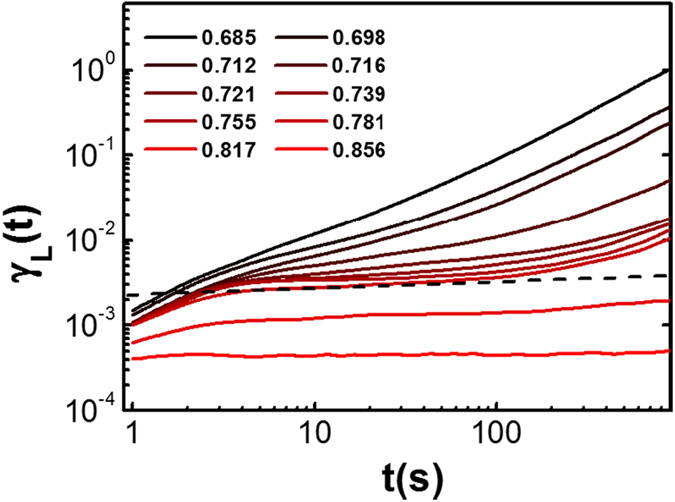
The dynamic Lindemann parameter, γ_L_ at different packing fractions. The Lindermann parameter stays finite in the crystal phase, and diverges in the hexatic and liquid phases (ϕ < 0.781). The dashed line is drawn to guide the eye, separating the finite regime from the diverging regime.

The dynamic Lindemann parameter, γ_L_ (t) is shown for different packing fractions (Fig. 5). The long time behavior can clearly distinguish the solid from the liquid phase. For the crystal phase (ϕ > 0.781), the Lindermann parameter γ_L_ stays finite, because the particles are caged by neighbors and vibrated about their lattice positions. For the hexatic and liquid phases (ϕ < 0.781), the Lindermann parameter γ_L_ diverges, as the particles can readily exchange their positions with neighbors. Here the solid-hexatic transition point is consistent with the result of translational susceptibility in Fig. 4.

### Defect dynamics

KTHNY theory predicts that there are pairings and separations of topological defects in 2D melting. Specifically the unbinding of a dislocation pair into free dislocations drives the solid-hexatic transition, and the unbinding of a disclination pair into free disclinations drives the hexatic-liquid transition. We take the advantage of our single-particles-level resolution experiments, the topological defects can be directly visualized in voronoi diagram. In our experiments, particles with *N* ≠ 6 are considered to be defects.

**Figure 6:**
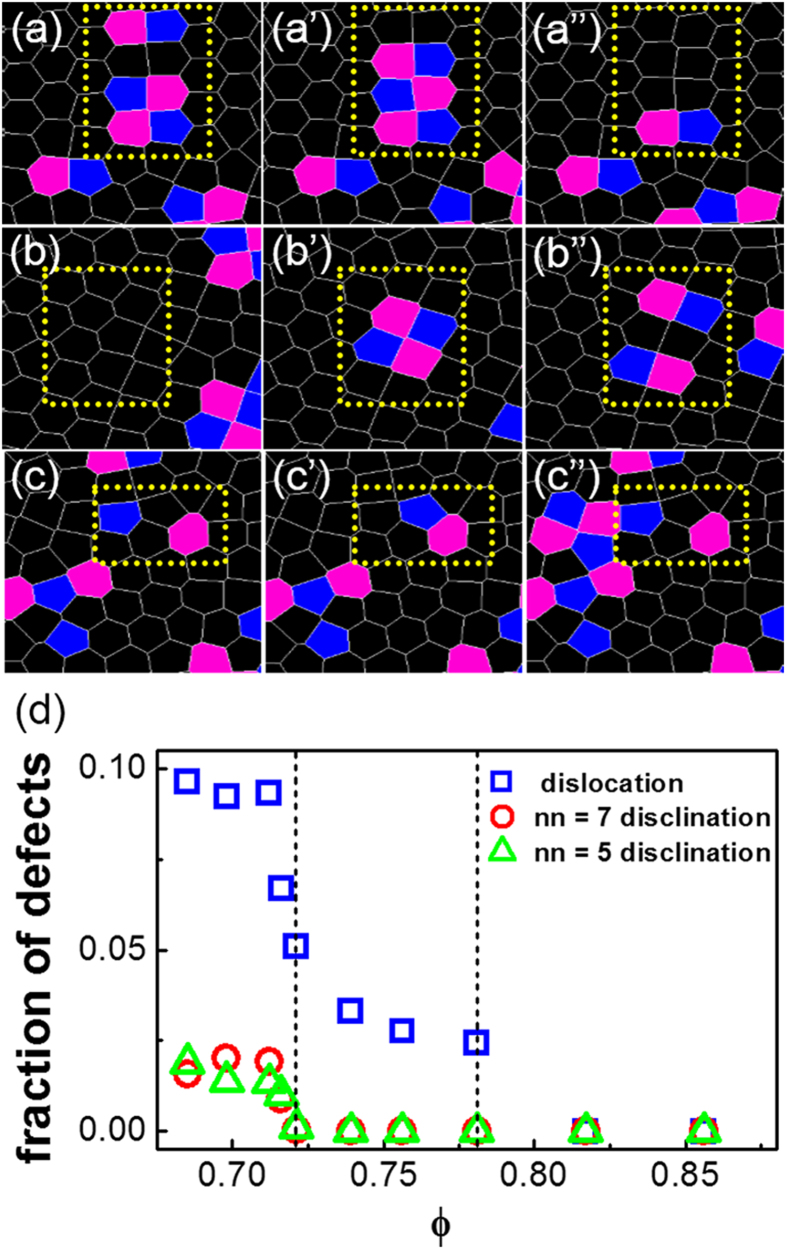
Defect dynamics and densities. (**a**–**c**) Time evolution of dislocation pairs 5-7-5-7 structure and declination pair 5–7 structure by Voronoi diagrams. Blue and pink represent particles with five and seven nearest neighbors, respectively. The yellow dashed rectangles highlight the evolutions. (**a**)–(a”) The formation and annihilation of dislocation pairs 5-7-5-7 structure. (**b**)–(b”) The appearance and separation of dislocation pairs 5-7-5-7 structure. (**c**)–(c”) The formation and separation of declination pair 5–7 structure. (**d**) The fraction of defects as a function of packing fractions.

The time evolution of dislocation pairs 5-7-5-7 structure and declination pair 5–7 structure in our system is shown in Fig. 6(a–c). When the system translates from the crystal to the hexatic phase, the dislocation pairs are aligned in opposite directions. When they are in the same lattice line, they will move together to form the 5-7-5-7 structure and then annihilated by fluctuation [Fig. 6(a–a”)]; and the process is reversible: the dislocation pairs can appear suddenly and then separate in the same lattice line [Fig. 6(b–b”)]^35^. When the system translates from the hexatic phase to the liquid, the isolated N = 5 or 7 defects can form 5–7 structure, and the 5–7 structure can also separate into the isolated N = 5 or 7 defects [Fig. 6(c–c”)]. Hence the topological defects shown in Fig. 6(a–c) are consistent with similar structures observed in 2D melting of colloidal systems^11^,^14^. The density of defects plot shown in Fig. 6(d) yield consistent results that dislocations start to appear for ϕ = 0.781, and disclinations start to appear for ϕ = 0.716.

## Summary

In summary, we have experimentally investigated the dynamical and structural behaviours of 2D melting of a model granular system using air-fluidized particles. We followed the melting process at single particle level with great spatial and temporal resolutions. In additional to conventional structural and dynamical quantities, a novel concept of orientational/translational susceptibilities was also applied to analyze the melting process. Using these methods, we resolved the intermediate hexatic phase, which was in accordance with the KTHNY theory, that the system followed a two-step melting, first from a crystal to a hexatic phase and then from a hexatic phase to a liquid, though the granular system is driven and athermal. Our results were consistent with previous work of 2D melting in colloidal systems and by computer simulations. Our work demonstrate further the applicability of driven granular systems to model behaviour in equilibrium systems, and provide a step forward in the quest for the nature of 2D melting.

## Methods

The air-fluidized granular apparatus under study here is originally developed by Durian’s group^36^,^37^. Plastic balls with a mean diameter of 3.12 ± 0.07 mm, are confined in a circular sieve with a diameter of 30 cm and a mesh size of 150 μm. The size of the circular boundary that confines the balls can be adjusted by using different machined inserts fitted in the sieve. A typical experimental system contains approximately three thousands balls. A variable transformer is used to control the air velocity^36^, which must be large enough to drive the balls to move smoothly and equally in all directions by turbulence, without levitation. Due to a fluidizing of air, the spheres can form a quasi-two-dimensional monolayer without slipping. In our experiment, we vary the number of particles to drive the melting process and define the packing fraction of the single layer as ϕ = *N*/*N*_max_, where *N* is the total number of particles in the field of view, and *N*_max_ is the maximum number of particles in a ‘static’ close packed layer. Here, the packing fraction is decreased from 0.856 to 0.685, and the air velocity is varied from 240 cm/s to 600 cm/s. The system is illuminated by four 25 W iridescent bulbs, which are arrayed in a 0.5 m diameter ring, located 1.5 m above the sieve. The reflected light from the top of each ball can be imaged by a high-speed digital camera (Prosilica), placed at the centre of the ring. The images are captured at a frame rate of 60 Hz, and streamed directly to a computer hard disk as AVI movies. The AVI movies are post-processed using ImageJ (NIH). For each frame the position of the ball can be identified with standard particle tracking algorithm^38^. Then the individual ball is tracked uniquely in the entire duration of experiment time. The tracking error in our study is ±0.08 mm, estimated by tracking the position fluctuation of a stuck ball.

## Additional Information

**How to cite this article**: Sun, X. *et al*. Direct observation of melting in a two-dimensional driven granular system. *Sci. Rep.* **6**, 24056; doi: 10.1038/srep24056 (2016).

## Supplementary Material

Supplementary Information
