# Supplementary material for: Direct observation of melting in a two-dimensional driven granular system
Source: Sci Rep. 2016 Apr 7;6:24056. doi: 10.1038/srep24056 (PMC4823733; doi:10.1038/srep24056)
Supplement: Supplementary Information [file srep24056-s1.pdf]

# Supplemental Material

# Direct observation of melting in a two-dimensional driven granular system

**Xiaoyan Sun [a,#] Yang Li [a,#] Yuqiang Ma [a,b,*] and Zexin Zhang[a,c,*]**

*[a] Centre for Soft Condensed Matter Physics and Interdisciplinary Research, Soochow University, Suzhou 215006, China. [b]National Laboratory of Solid State Microstructures and Department of Physics, Nanjing University, Nanjing 210093, China. [c] Kavli Institute for Theoretical Physics China, CAS, Beijing 100190, China*

** E-mail: myqiang@nju.edu.cn (Y. M.); zhangzx@suda.edu.cn (Z. Z.)*

## 1. The real space images of the air-fluidized granular system

We vary the number of particles to achieve desired packing fraction, and define the packing fraction of the single layer as $\phi = N/N_{max}$, where $N$ is the total number of particles in the field of view, and $N_{max}$ is the maximum number of particles in a 'static' close packed layer. In our experiments, the packing fraction is varied from 0.856 to 0.685, to drive the melting.

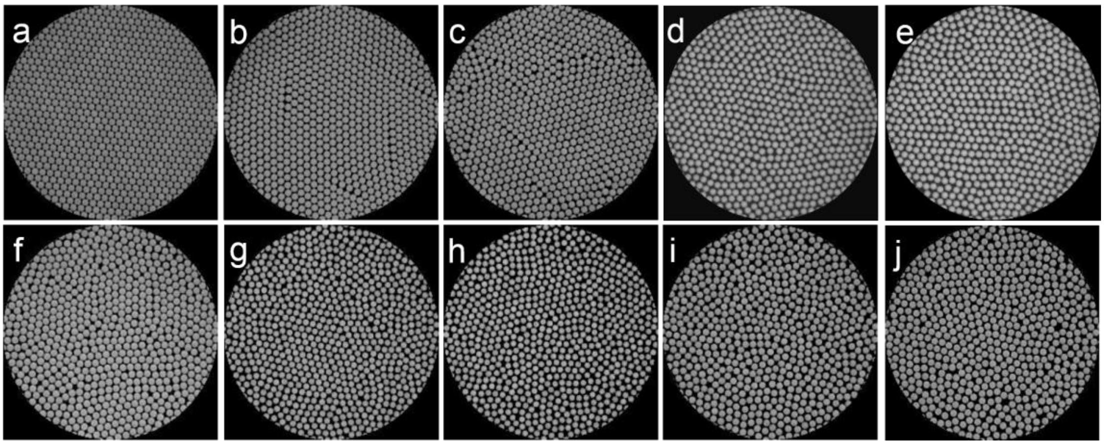


**Figure S1. Camera images of the air-fluidized granular system at different packing fraction.** From (a) to (j), the packing fraction is 0.856, 0.817, 0.781, 0.755, 0.739, 0.721, 0.716, 0.712, 0.698, and 0.685, respectively. Note that only one fourth of the field of view is shown for clarity.

**2. The extrapolation technique to obtain the susceptibility**

We divided the system into $n^2$ equal sub-boxes, and calculated the susceptibility for the each case. Then we use a second-order polynomial fit to extrapolate the susceptibility to $f(0)$ for the value at infinite system size. Note this extrapolation method has been used successfully to determine susceptibility in 2D melting by colloidal experiments and computer simulations [Han, Y. et. al. *Phys. Rev. E*, **77**, 041406 (2008); Chen, Y. *J. Chem. Phys.* **133**, 234508 (2010)].

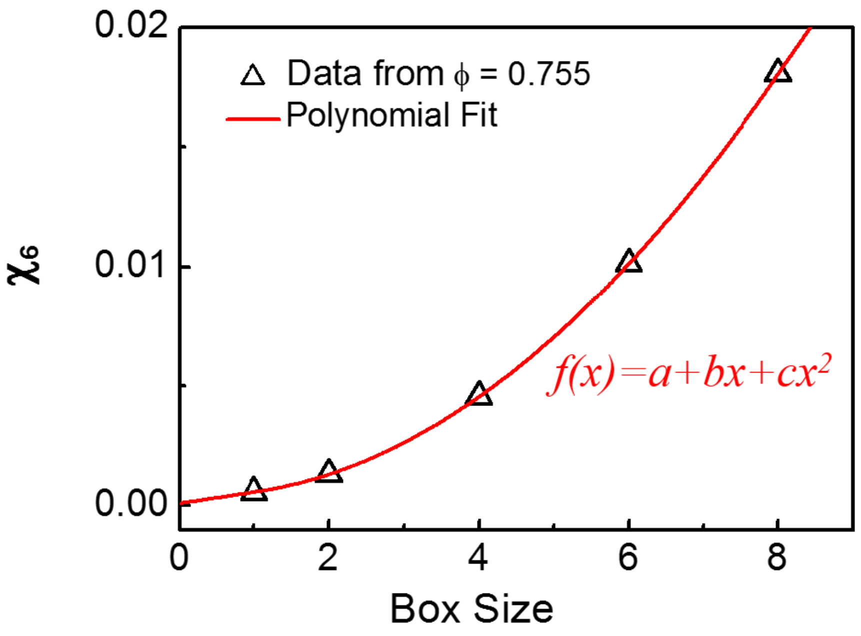


**Figure S2. Extrapolation method to obtain the susceptibility, $\chi_6$ for packing fraction $\phi$ = 0.755.** Here the box size, $n$ means that the system is divided into $n^2$ number of equal sized box, and the susceptibility for each case is calculated. The solid curve: $\chi_6$ for packing fraction $\phi$ = 0.755 fitted by $f(x) = a+bx+cx^2$, with $a = 4.09\times10^{-4}$, $b = -1.35\times10^{-4}$, and $c = 2.93\times10^{-4}$. The intercept of the polynomial fit on $y$ axis, $f(0) = a$, is taken as the susceptibility at the infinite size limit.
